# Osmostress-Induced Cell Volume Loss Delays Yeast Hog1 Signaling by Limiting Diffusion Processes and by Hog1-Specific Effects

**DOI:** 10.1371/journal.pone.0080901

**Published:** 2013-11-20

**Authors:** Roja Babazadeh, Caroline Beck Adiels, Maria Smedh, Elzbieta Petelenz-Kurdziel, Mattias Goksör, Stefan Hohmann

**Affiliations:** 1 Department of Chemistry and Molecular Biology, University of Gothenburg, Göteborg, Sweden; 2 Department of Physics, University of Gothenburg, Göteborg, Sweden; 3 Centre for Cellular Imaging, University of Gothenburg, Göteborg, Sweden,; Texas A&M University, United States of America

## Abstract

Signal transmission progresses via a series of transient protein-protein interactions and protein movements, which require diffusion within a cell packed with different molecules. Yeast Hog1, the effector protein kinase of the High Osmolarity Glycerol pathway, translocates transiently from the cytosol to the nucleus during adaptation to high external osmolarity. We followed the dynamics of osmostress-induced cell volume loss and Hog1 nuclear accumulation upon exposure of cells to different NaCl concentrations. While Hog1 nuclear accumulation peaked within five minutes following mild osmotic shock it was delayed up to six-fold under severe stress. The timing of Hog1 nuclear accumulation correlated with the degree of cell volume loss and the cells capacity to recover. Also the nuclear translocation of Msn2, the transcription factor of the general stress response pathway, is delayed upon severe osmotic stress suggesting a general phenomenon. We show by direct measurements that the general diffusion rate of Hog1 in the cytoplasm as well as its rate of nuclear transport are dramatically reduced following severe volume reduction. However, neither Hog1 phosphorylation nor Msn2 nuclear translocation were as much delayed as Hog1 nuclear translocation. Our data provide direct evidence that signaling slows down during cell volume compression, probably as a consequence of molecular crowding. Hence one purpose of osmotic adaptation is to restore optimal diffusion rates for biochemical and cell biological processes. In addition, there may be mechanisms slowing down especially Hog1 nuclear translocation under severe stress in order to prioritize Hog1 cytosolic targets.

## Introduction

Upon hyperosmotic stress water flows out of the cell resulting in an almost immediate decrease in cell volume and consequently an increase in the concentrations of all substances present in the cytoplasm. In order to recover turgor pressure, an appropriate cell volume as well as an internal water concentration optimal for biochemical processes, cells must adapt their internal osmolarity [1,2]. In this work we provide direct evidence that cell volume reduction delays signal transduction at least partly by strongly limiting the diffusion of a protein kinase. Therefore, an important aim of osmoadaptation appears to be re-establishment of an intracellular milieu compatible with diffusion rates required for cellular processes.

**Figure 1:**
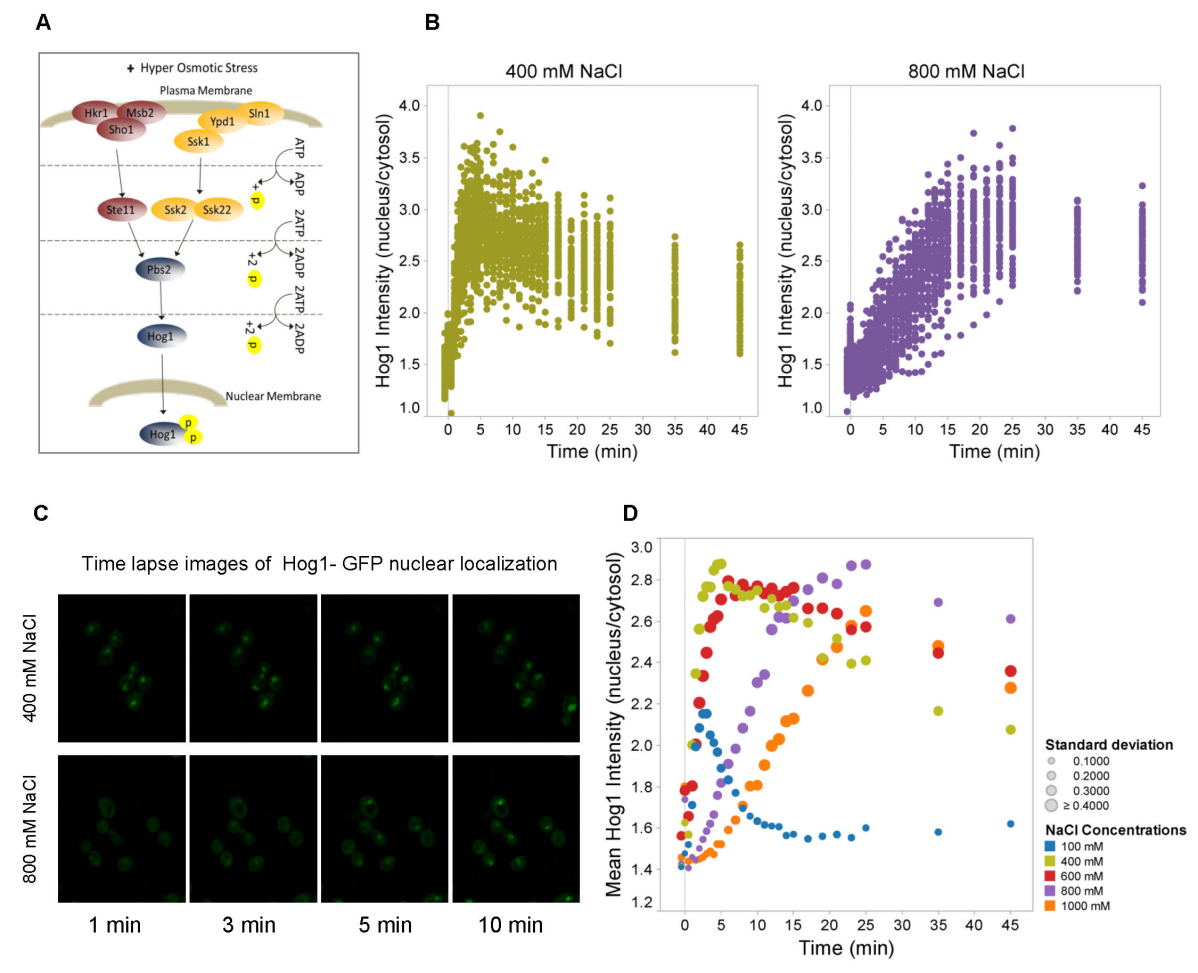
Nuclear accumulation of Hog1 is delayed under severe hyperosmotic stress. **A**. Scheme of the HOG signaling pathway. Upon hyperosmotic shock a branched cascade mediates dual phosphorylation and activation of Hog1. Phosphorylated Hog1 is then translocated into the nucleus where it associates with different DNA-binding proteins to mediate transcriptional regulation. **B**. Ratio of Hog1-GFP between nucleus and cytosol as a function of time in wild type cells. At time “0” the medium was adjusted to 400mM and 800mM NaCl, respectively. Data represent values for ca. 60 cells for each condition. **C**. Confocal time lapse images of the nuclear localization of Hog1 in 400mM and 800mM NaCl. Hog1 nuclear localization is delayed under severe osmotic condition (800mM NaCl). See Figure S2 for control data including Nrd1-mCherry, which marks the nucleus. **D**. Mean ratio of nuclear versus cytosolic Hog1-GFP of about 60 cells as a function of time upon different stress levels ranging from 100mM to 1,000mM NaCl. Colors symbolize the different salt concentrations and symbol sizes correspond to the standard deviation for each time point as indicated.

Similar to other eukaryotic cells, the yeast *Saccharomyces cerevisiae* responds to external stimuli via mitogen-activated protein kinase (MAPK) pathways [3-6]. High osmolarity activates the HOG MAPK signaling cascade (Figure 1A), which coordinates adaptive responses, such as a transient cell cycle arrest as well as accumulation of the compatible solute glycerol [7-9]. The Hog1 MAPK, the effector kinase of the HOG pathway, is activated by phosphorylation via the Sln1 and Sho1 upstream signaling branches, which converge on the Pbs2 MAPKK [1,4]. Phosphorylated Hog1 accumulates in the nucleus where it controls gene expression in collaboration with DNA-binding proteins such as Hot1 and Msn2 [10,11], which affect the expression of hundreds of genes [12-16]. 

Msn2 and Msn4 are partly redundant transcription factors necessary for transcription of many stress-induced genes, including those up-regulated by osmotic stress [17]. Hot1 appears to be the key transcription factor controlling glycerol production and uptake under osmotic stress [11,15]. Hot1 is involved in the control of expression of *GPD1* and *GPP2*, which encode the enzymes that convert glyceraldehyde-3-phosphate to glycerol [18-21]. There is a second isoform for glyceraldehyde-3-phosphate dehydrogenase encoded by *GPD2*, which acts under anaerobic conditions [22]; the double deletion mutant *gpd1∆ gpd2∆* cannot produce glycerol and is unable to adapt to hyperosmotic stress [22]. The intracellular glycerol content is also regulated via the glycerol channel Fps1, which mediates controlled export of excess glycerol [23]. Fps1 is regulated by Hog1 [9,23-25] but the mechanism is not fully understood.

Hog1 activity is tightly regulated because it is a negative regulator of cell cycle progression [8] and constitutively active Hog1 is lethal [26]. The phosphorylation state of Hog1 is controlled by different phosphatases including the protein tyrosine phosphatase Ptp2 and Ptp3 which are located in nucleus and cytosol, respectively [27,28]. Hog1 activation following hyperosmotic shock is transient [26,27,29,30] and different feedback control mechanisms have been reported [30-34], most notably successful osmoadaptation itself [35]. 

Hog1 nuclear accumulation has previously been employed as a read-out for HOG pathway activity. Those studies addressed a range of different questions including the intracellular distribution pattern of Hog1 [36], the negative feedback that ensures perfect system adaptation [34], osmoadaptation mechanisms independent of nuclear Hog1 function [37], the transcriptional behavior and thresholds in response to osmotic stress [38], signaling specificity between MAPK pathways [39] as well as characterizing the role of signal integration [40] and MAPK pathway communication upon different simultaneous stimuli [41].

It has previously been observed that severe osmotic shock leads to prolonged phosphorylation of Hog1 and a delayed induction of stress-responsive genes [1,42]. The delayed transcriptional response has been correlated with a defect in Hog1 translocation from the cytoplasm to the nucleus [42]. In this work we investigated the mechanism of the signaling delay by monitoring cell volume loss and recovery as well as Hog1 nuclear accumulation following different degrees of osmoshock. For this purpose we employed cell arrays in a microfluidic device mounted under a fluorescence microscope. This setting [43] allows optimal control of the cells’ environment and rapid and precise changes of the external osmolarity. We correlated Hog1 nucleo-cytoplasmic shuttling with cell volume changes and we monitored directly Hog1 diffusion rates and the rate of Hog1 nuclear transport under different conditions. Our data are consistent with a picture where strong osmostress causes cell volume compression below a threshold where molecular crowding may critically inhibit signal progression. In addition, by carefully comparing time courses, we suggest that Hog1 nuclear accumulation may be specifically delayed, perhaps in order to perform critical cytosolic functions. Our observations confirm and extend observations published while this manuscript was prepared [44]. Specifically, these authors reported osmostress-induced slow-down of protein diffusion, different signaling pathways as well as vesicular trafficking and they concluded that macromolecular crowding caused by cell compression can explain those observations.

## Materials and Methods

### Yeast strains and Media

To quantify Hog1 nuclear localization, Hog1-GFP and Nrd1-mCherry (“Nuclear pre-mRNA Down-regulation”; to mark the nucleus) fusion constructs were integrated into the genome of the BY4741 genetic background employing wild type as well as *gpd1∆ gpd2∆, fps1∆, ptp2∆* and *ptp3∆* mutants. Msn2-GFP and Nrd1-mCherry constructs were integrated into the genome of the BY4741 wild type for quantification of Msn2-GFP nuclear localization. Cells were grown in two-fold Yeast Nitrogen Base medium (YNB 1.7 g/L, ammonium sulfate 5 g/L) to exponential phase. Osmostress conditions were established in cell cultures by adding a stock solution of 5M NaCl to YNB medium to final NaCl concentrations of 100, 400, 600, 800, and 1000mM NaCl.

### Microfluidics and microscopy setup

Single cell experiments were performed using the previously described microfluidic systems with three inlet channels [43]. The upper inlet channel was used to introduce into the system cells suspended in growth medium. The middle channel was used for growth medium and the lower inlet channel for growth medium with NaCl. The flow rates of the different solutions could be controlled independently, enabling rapid and precise changes of the environment exposed to the cells, which were positioned in the measurement region close to the junction of the inlet channels.

Lectin-treated microfluidic systems (1 mg/ml concanavalin A, 10mM Tris-HCl, 100mM NaCl, pH 8.0) were placed on the stage of an inverted epi-fluorescence microscope. The microscope (DMI6000B Leica Microsystems), a 14 bit dynamic range EM-CCD camera (C9100-12, Hamamatsu Photonics) and a set of CMA/400 syringe pumps (Microdialysis AB) were all controlled using custom written scripts in the OpenLab Automator extension of OpenLab (Improvision Inc.) software. This enabled control of the extracellular environment during simultaneous time lapse imaging. 

Individual yeast cells were trapped in the liquid flow using a 1070 nm ytterbium fiber laser (YLD-5-LP, IPG Laser) and positioned into the measurement region inside the microfluidic system. During the positioning of the cells on the surface, the flow rate in the lower inlet channel was set to 40 nl/min, while the flow rates in the two other channels were set to 80 nl/min. Thereafter the flow rates were switched to 1000 nl/min in the lower inlet and completely stopped in the other two inlets to expose cells to the osmotic stress solution. 

To measure the dynamic shuttling of Hog1-GFP or Msn2-GFP between the nucleus and cytosol as well as the cell volume recovery axial stacks (spacing 0.8 µm) both fluorescence and bright field images were acquired using a 100 x HCX plan fluotar oil immersion objective. Images were taken sequentially every 30 sec for 300 sec, every 60 sec for 600 sec, every 120 sec for 600 sec and every 600 sec for 1200 sec thus yielding a total experiment period of 45 min. The nuclei were detected using the nucleus-resident reporter Nrd1-mCherry and the images were analyzed using CellStress software [45].

### Protein extraction and Western blotting

Cells were collected and re-suspended in sodium dodecyl sulfate (SDS) loading buffer (100mM Tris-HCl [pH 6.8], 200mM dithiothreitol, 4% SDS, 20% glycerol, 20mM mercaptoethanol, 10mM NaF, 0.1mM Na-vanadate, protease inhibitor [complete EDTA-free protease inhibitor cocktail tablets; Roche]). Cell suspensions were boiled for 10 min and then centrifuged at 13,000 x g at 4°C for 10 min to obtain protein extracts. 50μg of protein was separated on an SDS (10%) polyacrylamide gel and blotted onto nitrocellulose membranes (Hybond TM ECL TM, Amersham). Membranes were blocked with Odyssey blocking buffer for 1 h and incubated overnight at 4°C with the anti-phospho-p38 MAPK primary antibody (Thr180/Tyr182; Cell Signaling) and total anti-Hog1 (yC-20, Santa Cruz), respectively, diluted 1:1000 in blocking buffer (0.1% Tween 20). After washing (4x5 min in TBS-T), membranes were incubated with IRDye 680 donkey anti rabbit and IRDye 800 donkey anti goat (LI-COR Biosciences) secondary antibodies diluted 1:15,000 in blocking buffer for 1 h at room temperature. The washing step was repeated before scanning (Odyssey scanner, Li-Cor Biosceinces).

### Quantitative PCR

Total RNA was isolated using the RNeasy®Mini Kit (Qiagen) and treated with DNase (RNase-Free DNase Set (Qiagen). Subsequently, cDNA was synthesized from 0.8μg purified RNA using anchored oligo-dT primer (ABgene) and SuperScript^TM^ II transcriptase (Invitrogen), following instructions from the manufacturer. The Quantitative PCR reaction was performed in an iQ5 iCycler (Bio-Rad) with 5 μl of cDNA, 10 ul of iQ SYBR Green supermix I PCR (Bio-Rad) and 10 μM of specific primers for *ALD2* (5′-GTTGCAGGAAAATTTGATCCG -3′ and 5′- AGAACGTGGTAAAAGGGAGGA -3′). Expression data were normalized against the housekeeping genes *ACT1* (5′-ACCGCTGCTCAATCTTCTTC-3′ and 5′-ATGATGGAGTTGTAAGTAGTTTGG-3′) which encodes actin. The following amplification conditions of were applied: 3 min at 95 °C; 40 cycles of 10 sec at 95 °C, 30 sec at 58 °C; 81 cycles of 8 sec at 55 °C.

### FCS

Fluorescence correlation spectroscopy (FCS) measurements [46] were performed on a ConfoCor2 FCS unit attached to an inverted LSM 510 Meta confocal microscope (Carl Zeiss), using a C-Apochromat 40x/1.20 W Corr objective. In order to find the measurement position in the cytoplasm of each cell GFP (excited at 488 nm and emission detected between 500-530 nm), mCherry (excited at 543 nm and emission detected above 560 nm) and the transmission channel were simultaneously imaged using an open pinhole. The measurements were performed for 10 x 8 sec with excitation using 488 nm and detection at 500-550 nm, pinhole 1 AU. The position of the FCS measurement versus the LSM image was calibrated using a fluorescent glue (Kleber 44, Carl Zeiss) sample and marked by a cross hair in the image. Cells were attached to a ConA treated cover glass and 12 cells were analyzed for both stress and non-stress conditions. The measurements was started 2.5 min after stimulation with 400mM or 800mM NaCl. 

The power of the 488 nm laser was set to an AOTF transmission of 0.02 % in order to avoid any visible bleaching while maintaining a sufficient signal to noise ratio for untreated cells. However, since the processes were slower in NaCl-treated cells these might be more prone to bleaching and hence the measured diffusion times cannot be directly associated to diffusion coefficients. Laser power could not be decreased further due to low fluorescence signal. However, bleaching usually causes the diffusion time to get shorter. Therefore, the actual diffusion times for the NaCl-treated cells should be at least as slow as or slower than the diffusion times measured here. Since NaCl-stressed cells also had a smaller volume the possible influence of measuring in a restricted compartment might be greater. 

Fitting of the FCS autocorrelation curves was done using a single component free diffusion fit in the ConfoCor2 software. The normalization and averaging of single cell data was done in MATLAB (The MathWorks, Inc.).

### FRAP

Flourescence recovery after photobleaching (FRAP) experiments [47] were performed on Hog1-GFP in BY4741 by bleaching the nuclear region and monitoring the recovery using an inverted LSM 700 confocal microscope (Carl Zeiss). Nrd1-mCherry was used as a nuclear marker to set the nuclear bleach region correctly. GFP (excited at 488 nm and emission detected below 555 nm), mCherry (excited at 555 nm and emission detected above 575 nm) and a transmission channel where simultaneously imaged using a Plan-Apochromat 63x/1.40 oil objective and a pinhole setting of 1 AU for the fluorescence channels. The frame time was 97.75 ms using zoom factor 6 and 256×256 pixel resolution (pixel dwell time 1.27 µsec). The FRAP time series consisted of a total of 100 scan cycles, with a photo bleaching event after 5 scans. Cells were attached to a ConA treated cover glass and 15 cells were analyzed for each stress condition, stimulated either by 400mM or 800mM NaCl for 2.5 min and one free-hand nuclear bleach region per image. The fitting of the recovery curves and the averaging of single cell data were done in MATLAB (The MathWorks, Inc.).

## Results

### Nuclear accumulation of Hog1 is delayed under severe hyperosmotic stress

To study the response characteristics of the HOG pathway in real time, we monitored the dynamics of Hog1-GFP nucleo-cytoplasmic shuttling in individual yeast cells under osmotic stress conditions in the presence of 100, 400, 600, 800 and 1000mM NaCl. Ca. 30 cells per run were picked using optical tweezers and arranged as a cell array in a microfluidic device to rapidly and precisely change environmental conditions. Data at any given time point (Figure 1B) are presented as the ratio of nuclear to cytoplasmic fluorescence intensity for each individual cell. Hog1 accumulated in the nucleus rapidly and transiently, similar to what has been reported previously [34,42,44]. Although there seems to be cell-to-cell variability in terms of the peak intensity of the response the overall response profile of individual cells appeared to be very similar under the conditions tested. 

The Hog1-GFP nuclear accumulation profile was dependent on the degree of osmotic stress. At lower stress levels (100 and 400mM NaCl) the peak intensity of the response increased with the stress level while at progressively higher stress levels the period of Hog1 nuclear residence was extended. At very high stress levels like 800mM or 1000mM NaCl, Hog1 nuclear migration was clearly delayed (Figure 1C, Figure S1). While Hog1 nuclear accumulation peaked after 3 min in 100mM NaCl, the peak intensity was reached after 5 min in 400mM NaCl, and after approximately 6, 15, and 25 minutes in 600, 800, and 1000mM NaCl, respectively (Figure 1D). 

We repeated Hog1 nuclear accumulation profiling in a *gpd1∆ gpd2∆* mutant, which is unable to produce glycerol and hence cannot adapt to high external osmolarity [22,48]. While these cells are initially still alive (but cannot adapt) at even the highest NaCl concentration (data not shown), Hog1 did not localize to the nucleus within the period of the time lapse experiment (45min) when cells were treated with 1000mM NaCl and only slowly reached a plateau much lower than the peak intensity observed for wild type. Possibly, the plateau increases even beyond the measurement for the 800 mM NaCl treatment (Figure S2). Taken together and consistent with previous reports [49-51], the Hog1 nuclear accumulation profile is strongly affected in cells with impaired capacity to accumulate glycerol and adapt to the stress. 

### Hog1 nuclear accumulation correlates with cell volume recovery dynamics

**Figure 2:**
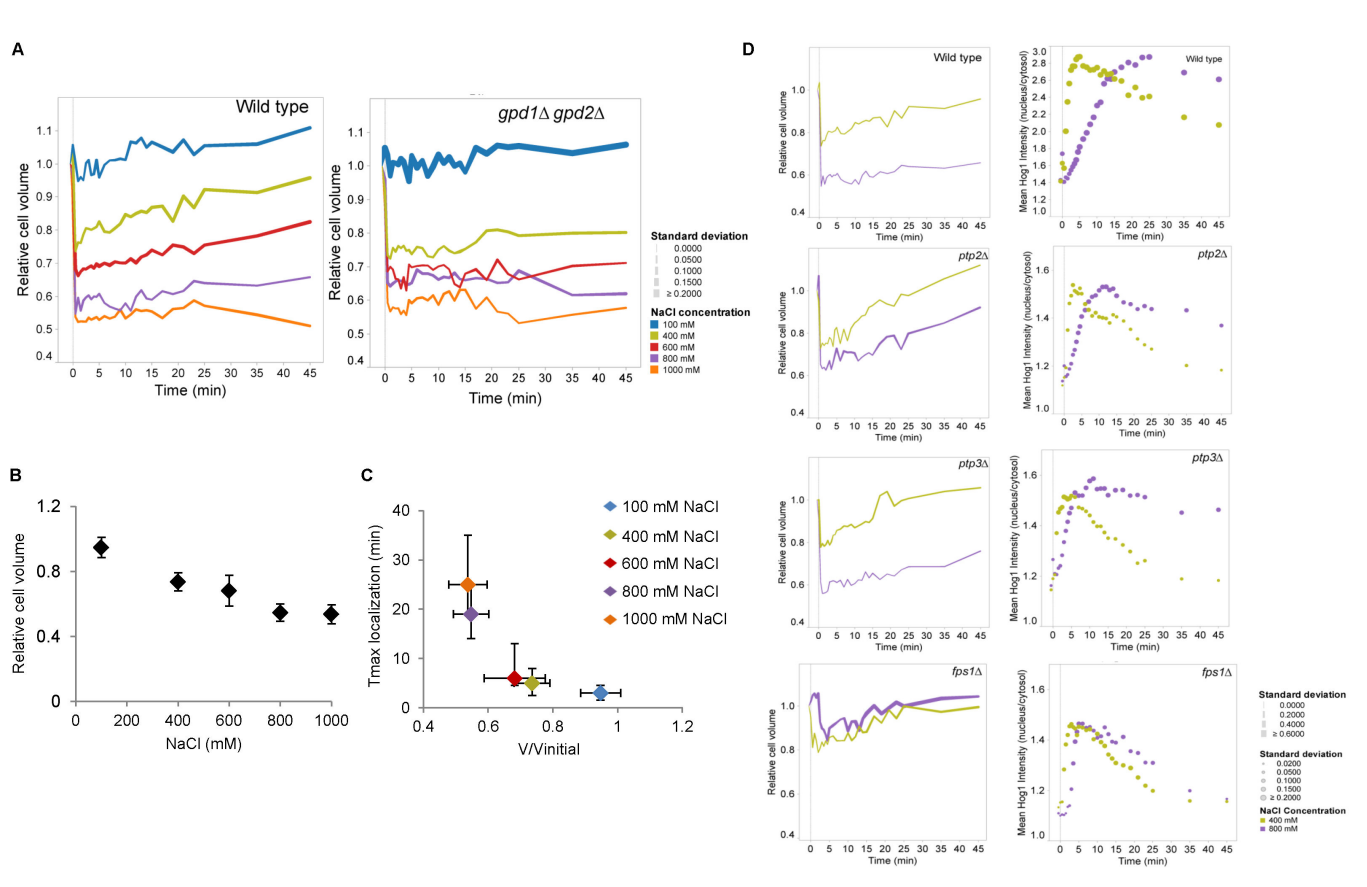
Hog1 nuclear accumulation correlates with cell volume recovery dynamics. **A**. Relative cell volume changes of cells treated with different concentrations of NaCl in wild type and the *gpd1∆* *gpd2∆* mutant. Colors indicate different salt concentrations and symbol sizes show the standard deviation for each time point. Ca. 60 cells were monitored. **B**. Relative cell volume (untreated cells = 1.0) as a function of NaCl concentration in wild type cells. Data represent the average of about 60 cells and the error bars indicate the standard deviation between cells. Cell volume was monitored over time and for each cell the lowest volume value at the relevant salt concentration was used to calculate the average. **C**. The time point at which Hog1 nuclear concentration reaches its maximum as a function of relative cell volume compression. Data on the y-axis represent the average time point of maximal Hog1 nuclear localization of about 60 cells and the vertical errors bars present the variation between the time points in which cells reach their Hog1 maximum localization. Data on the x-axis represent maximal relative cell volume reduction of those cells and the horizontal bars the standard deviation between cells. **D**. Changes of relative cell volume (left panels) and Hog1-GFP nuclear localization (right panels) upon treatment with 400mM and 800mM NaCl in wild type, *ptp2∆*, *ptp3∆*, and *fps1∆* mutants. Colors represent the different salt concentrations and symbol sizes indicate the standard deviation for each time point. Data represent about 60 cells for wild type and ca. 30 cells for each mutant.

In order to relate Hog1 nuclear accumulation dynamics with cell volume changes, we examined volume loss and recovery over time in response to 100, 400, 600, 800 and 1000mM NaCl in wild type and *gpd1∆ gpd2∆* mutants. The cell volume dropped immediately after osmotic shock (Figure 2A) and volume reduction displayed an approximately linear relationship with salt concentration (Figure 2B). Cells gradually restored their volume over time but the rate of recovery differed significantly between mild and severe stress. For instance, in 400mM NaCl, wild type cells lost on average 30% of their initial volume, which they almost completely regained after 45 minutes. Cells exposed to 800mM NaCl lost approximately 55% of their initial volume, of which only 10% were recovered within 45 minutes (Figure 2A, B). 

We correlated volume reduction with the period until nuclear accumulation of Hog1 reached its peak intensity (Figure 2C). The apparent exponential correlation between the Hog1 nuclear translocation rate and the volume changes clearly illustrates that cellular compression slows down Hog1 nuclear accumulation. To study the basis for this delay further, we compared the timing of Hog1 nuclear localization and cell volume recovery in *ptp2∆*, *ptp3∆*, and *fps1∆* strains upon 400mM and 800mM NaCl treatment (Figure 2D). Ptp2 and Ptp3 are two protein phosphatases that dephosphorylate Hog1 in the nucleus and cytosol, respectively, and deletion of *PTP2* but not of *PTP3* increases basal Hog1 activity [30,52]. Fps1 encodes the plasma membrane glycerol channel and its activity is inhibited by Hog1 [9,25,53]. Deletion of *FPS1* causes an increase in basal cytosolic glycerol levels [23,24,54,55]. We observed that in cells treated with 800mM NaCl, Hog1 nuclear localization occurred faster in all three deletion strains compared to wild type (Figure 2D). We further observed that *ptp2∆* and *fps1∆* cells stressed with 800mM NaCl lost less of their volume and regained their cell volume more efficiently. Hence, genetically “pre-adapted” cells appear to recover their volume faster and also show faster Hog1 nuclear accumulation. These findings further confirm a strong connection between the timing of Hog1 nuclear localization and the cell’s capacity to restore its volume.

### Nuclear accumulation of stress-responsive Msn2-GFP is delayed in severe hyper-osmotic stress

**Figure 3:**
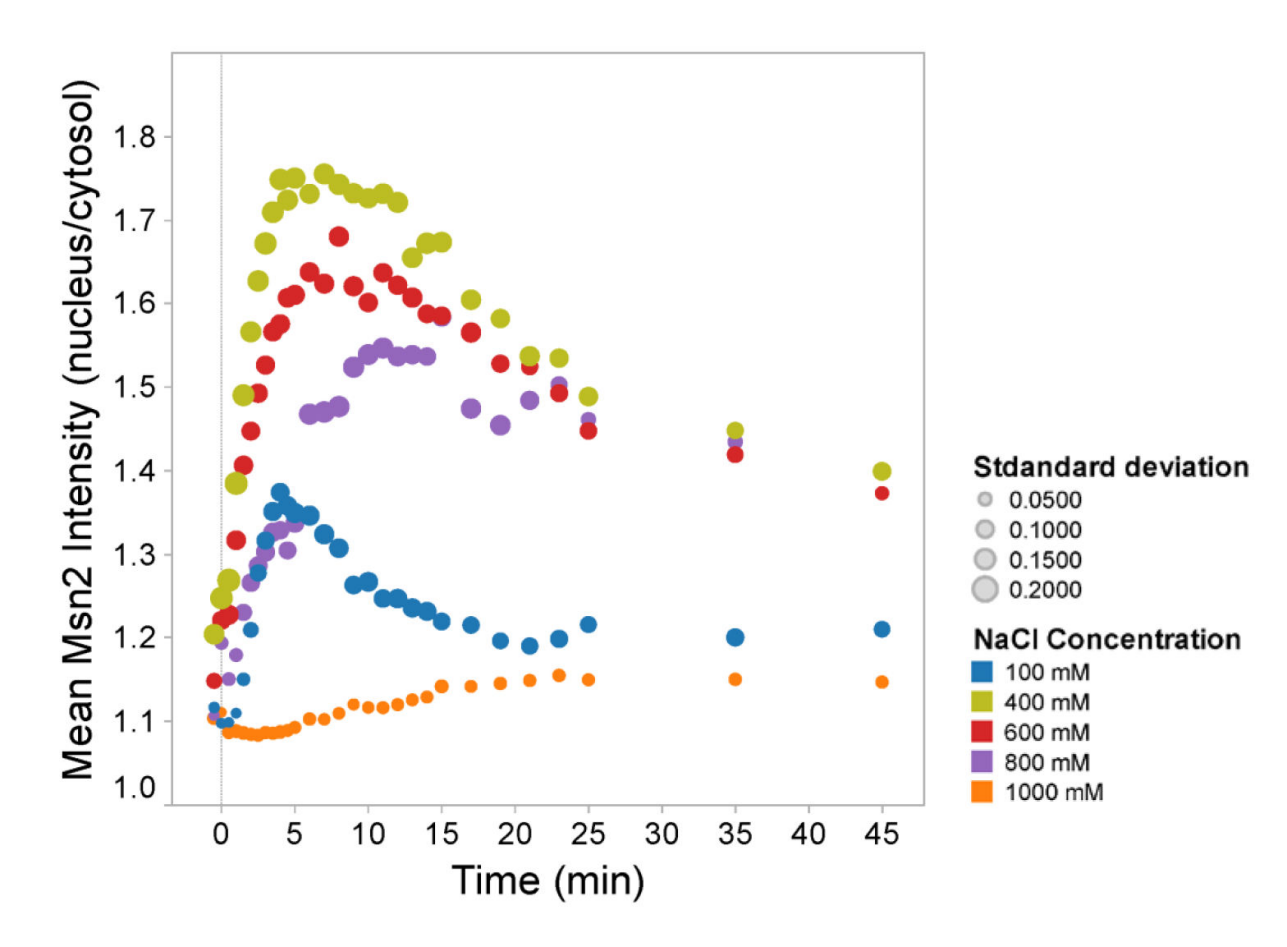
Nuclear accumulation of stress-responsive Msn2-GFP is delayed in severe hyper-osmotic stress. Mean ratio of nuclear versus cytosolic Msn2-GFP as a function of time for different stress levels in wild type. Colors represent different salt concentrations and symbol sizes indicate the standard deviation for each time point.

To investigate whether the decrease in nuclear accumulation rate upon strong osmotic stress is specific for Hog1 or if other proteins involved in the osmotic response follow the same pattern, we investigated nuclear translocation of Msn2. Msn2 is, together with its paralog Msn4, the key transcription factor of the general stress response pathway. Msn2 migrates to the nucleus in response to different stress conditions, including hyper-osmotic stress, and nuclear accumulation is controlled by the PKA pathway as well as a different nuclear import mechanism than Hog1 [36,56,57]. We observed that similar to Hog1-GFP translocation the peak intensity of the Msn2-GFP nuclear/cytoplasmic ratio occurs within 3 minutes after treatment with 100mM NaCl and 5 minutes after cells have been exposed to 400mM NaCl (Figure 3). However the Msn2-GFP nuclear peak in 600mM and 800mM NaCl was reached faster than that of Hog1, i.e. after 8 and 10 minutes, respectively. Following treatment with 1000mM NaCl, the nuclear localization of Msn2 only slightly increased after 20 minutes. Taken together, although Msn2 nuclear localization was not as severely delayed as that of Hog1 it appears that the observed signaling delay is a general phenomenon under severe osmostress. We note, however, that it has been reported that Hog1 partly affects the degree of osmostress-induced nuclear accumulation of Msn2 [40].

Since Msn2 nuclear localization is dramatically diminished in 1000mM NaCl, we investigated whether cells can trigger an Msn2/4-dependent transcriptional response at high NaCl levels. We chose *ALD2* expression as Msn2 and Msn4 target [15] and measured the *ALD2* mRNA level in wild type as well as in *msn2∆* mutants treated with 400 and 1000mM NaCl and in *msn2∆ msn4∆* cells treated with 400mM NaCl. As expected, *ALD2* expression was completely dependent on Msn2/4 while the single *msn2∆* mutant still showed a similar temporal pattern of expression as the wild type, albeit with lower peak intensity (Figure S3). As previously reported [15,42], *ALD2* upregulation in wild type and *msn2∆* cells occurs later at higher salt concentration (Figure S3). In conclusion, hyper-osmotic stress does not seem to abolish but rather delay Msn2/4-dependent transcriptional activity, mirroring the delayed nuclear translocation of the transcription factor. The apparently lower nuclear Msn2 levels still seem to be sufficient to stimulate a strong transcriptional response.

### Hog1 phosphorylation is delayed in severe hyper-osmotic stress

**Figure 4:**
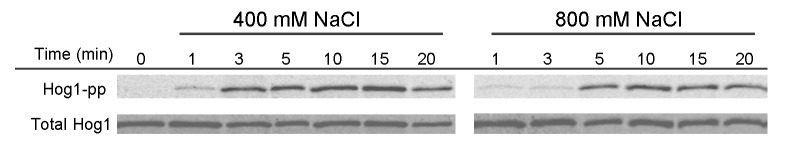
Hog1 phosphorylation is delayed in severe hyper-osmotic stress. Western blot of Hog1 phosphorylation in wild type treated with 400mM and 800mM NaCl at time “0”. The upper blot was treated with antibody recognizing dually phosphorylated Hog1, the lower panel with an antibody that detects total Hog1.

In order to investigate the mechanism underlying the delayed Hog1 nuclear accumulation under extreme hyper-osmotic stress, the dynamics of Hog1 phosphorylation were studied following treatment with 400mM and 800mM NaCl (Figure 4). Phosphorylation of Hog1 is a prerequisite for its accumulation in the nucleus [57]. After treatment with 400mM NaCl, Hog1 phosphorylation reached its maximum level within the first 5 min, whereas at 800mM NaCl Hog1 phosphorylation peaks after 10 min. Hence, although Hog1 phosphorylation is delayed in very high salt concentration, the delay appears to be shorter and hence does not completely explain the even slower Hog1 nuclear accumulation under these conditions.

### Free diffusion of Hog1 in the cytoplasm is strongly reduced in osmostressed cells

**Figure 5:**
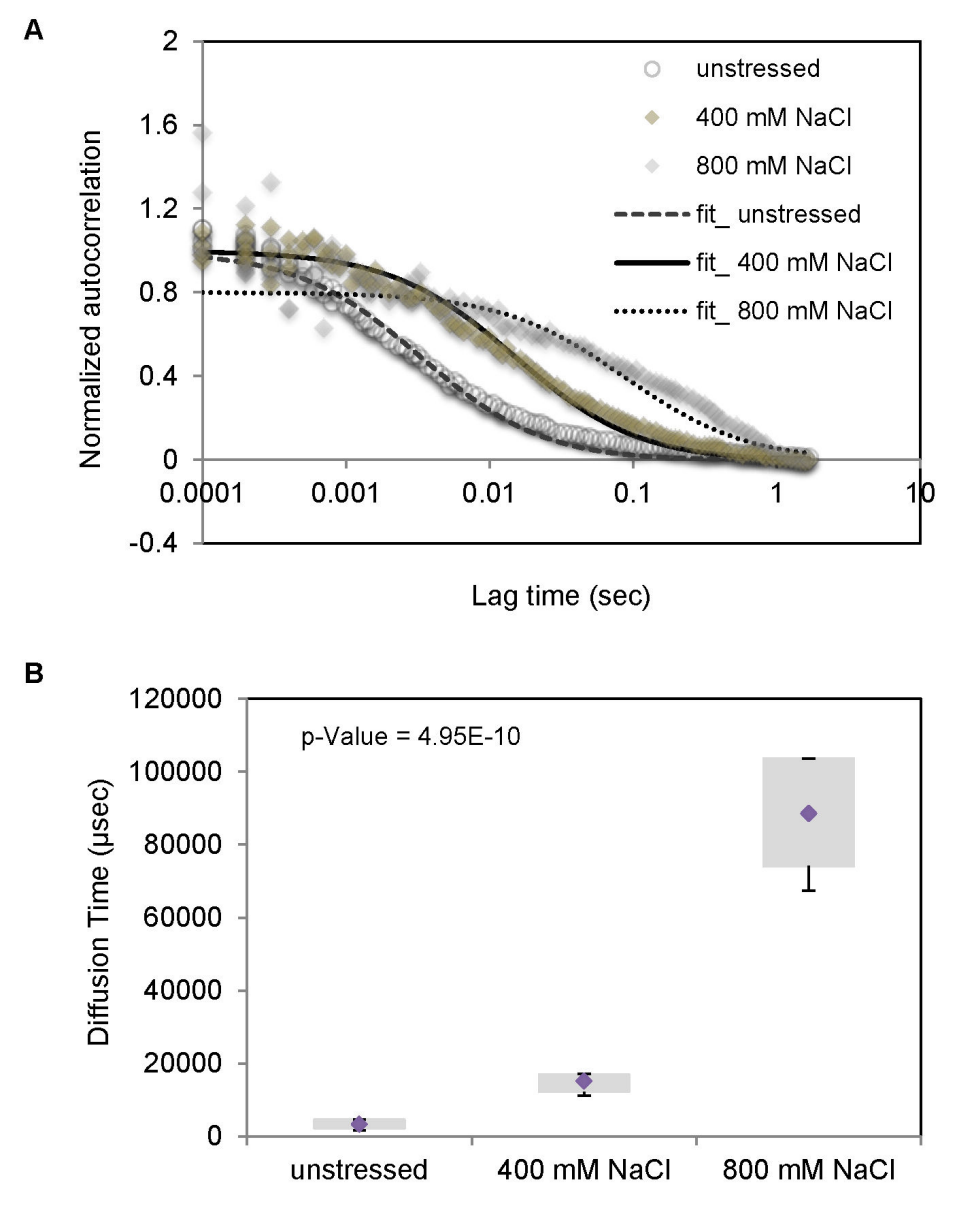
Free diffusion of Hog1 in the cytoplasm is strongly reduced in osmo-stressed cells. **A**. The average FCS autocorrelation curves of 12 wild type Hog1-GFP, Nrd1-mCherry cells in the absence of stress and in the presence of 400mM, and 800mM NaCl media. **B**. Hog1-GFP diffusion time for wild type as obtained from the fits of the data represented in (A) in the absence of stress as well as in cells treated with 400mM, and 800mM NaCl, respectively. The bottom and top of the box represent the first and third quartiles. The diamond shows the mean and whiskers indicate the variability of diffusion times outside the upper and lower quartiles for the 12 cells. Measurements were performed 2.5 minutes after cells were treated with salt.

In order to further investigate the mechanisms causing the HOG signaling delay we monitored directly Hog1 diffusion in the cytoplasm by fluorescence correlation spectroscopy (FCS) employing a wild type strain in the absence and presence of stress. FCS allows studying intensity fluctuations due to Brownian motions of fluorescent molecules in a very small detection volume inside living cells [46]. Hence, FCS data provide direct information about the averaged diffusion time at single-molecule level. Cells were treated with 400mM and 800mM NaCl for 2.5 min before measurements were started. Data were treated by normalized autocorrelation (Figure 5A), clearly indicating different diffusion rates under different conditions. The measured diffusion time (Figure 5B) showed that the diffusion dynamics of Hog1 is decreased with increasing osmotic concentration. Hog1 diffusion rates are diminished about 5-fold in the presence of 400mM and about 25-fold at 800mM NaCl (Figure 5B). Hence, the delay of Hog1 nuclear accumulation might partly depend on a strongly diminished free diffusion time in cytoplasm.

### The Hog1 nuclear import rate is similar for cells in different osmotic stress conditions

**Figure 6:**
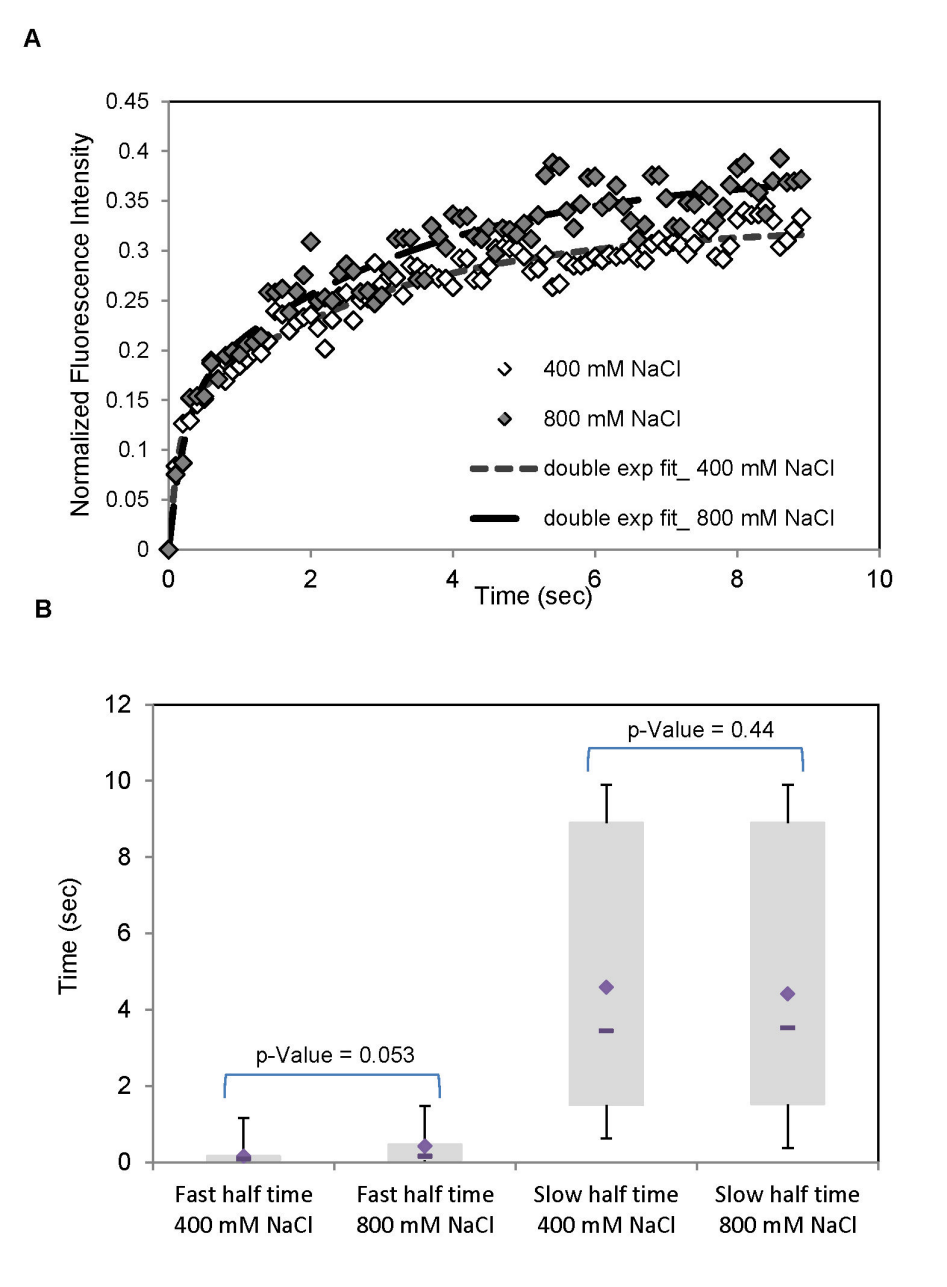
The Hog1 nuclear import rate is similar for cells in different osmotic stress conditions. **A**. FRAP (Fluorescence Recovery After Photobleaching) experiments on Hog1-GFP (Nrd1-mCherry as nuclear marker) in wild type cells to measure the rate of Hog1 nuclear import under two different osmostress conditions, 400mM and 800mM NaCl. The recovery curves, i.e. the mean intensity in a nuclear bleached region as a function of time, represent the average of the individual GFP-recovery curves for 15 cells. All measurements were performed after cells were treated with salt for 2.5 minutes. Subsequently, the area of the nucleus was bleached and the times on the x-axis represent the period after which the measurements were started. The recovery curves are fitted with a double exponential fit. **B**. Box plots for the fast and slow recovery half times from double exponential fits for 400 and 800 mM NaCl. The bottom and top of the boxes present the first and third quartiles. The diamond and dash line show the mean and median respectively. Whiskers indicate the variability of recovery half times outside the upper and lower quartiles. The data are consistent with two different mechanisms of Hog1 nuclear import under osmostress, a slow and probably passive mechanism as well as a fast and probably active mechanism.

To study delayed Hog1 nuclear accumulation further, fluorescence recovery after photo bleaching (FRAP) experiments were performed in order to directly compare the Hog1 nuclear import rates under different osmotic stress conditions [47]. Cells were treated with 400mM and 800mM NaCl for 2.5 min, the nucleus was bleached and fluorescence recovery was recorded over 10 seconds. The recovery curves were not possible to fit with single exponential curves. Instead we had to use a fit with double exponentials which suggest that there are at least two mechanisms for Hog1 nuclear transport, a slow and a fast one. The slow transport may correspond to a passive mechanisms and the fast transport to an active one. The recovery curves appears to be very similar following both 400mM and 800mM NaCl treatment (see Figure 6), which implies that the nuclear import rate does not play a major role for the delayed nuclear accumulation at higher osmotic stress. However, while the median value of the rate of the slow Hog1 nuclear import mechanism is almost identical ( 3.5 s) the fast import rate is almost three times slower in cells treated with 800mM NaCl (median value 0.47 s), as compared to cells treated with 400mM (median value 0.16 s) (Figure 6B). The estimated contribution to the nuclear import via the fast and slow process respectively is similar in size, with a median value of ~50% for both stress conditions, and therefore not negligible. Hence, the FRAP data suggests that the rate for Hog1 nuclear import might be impaired at short time scales under strong osmotic stress, which could have an effect on the observed delay in Hog1 nuclear accumulation. However, the differences are not statistically significant for this limited data set. The slower accumulation of Hog1 into the nucleus might hence be a combination of different effects, i.e. slower Hog1 diffusion in the cytoplasm, slower nuclear import and/or a cytosolic anchor mechanism acting on Hog1 under strong osmostress.

## Discussion

We provide evidence that cell volume reduction by osmotic stress results in a slow-down of cellular diffusion processes. The most obvious explanation for this phenomenon is molecular crowding: cellular volume reduction caused by osmotic stress is to a very large degree due to water loss and hence leads directly to an increase in the concentration of all molecules inside the cell. Given the normally already high concentration of proteins and other molecules a reduction in cell volume by for instance 30% may then result in concentrations that are critical for the movement of molecules such that the rate of cellular processes is reduced. Such a volume reduction is observed following treatment with 400mM NaCl and caused an about 5-fold lower cytosolic Hog1 diffusion rate. This in turn means that the homeostatic concentrations of molecules in the cell are held rather close to such critical levels and compression of the cell by for instance 30% significantly affects intracellular processes. The observed phenomena encompass Hog1, Msn2 (this work and [44]), Mig1, Yap1 and Crz1 [44] nuclear localization, as well as vesicular trafficking [44]. In this work we provide direct evidence by FCS performed on wild type cells that Hog1 diffusion in the cytoplasm is restricted, most probably because of cell compression following osmotic shock. In this way we strengthen and extend the conclusions of [44], where diffusion rates were measured by FRAP (which is not optimal to determine diffusion coefficients since the broad bleaching area introduces large errors) and where the use of a *pbs2* mutant may have caused further confounding effects. It has always been stated that osmotic adaptation among other things aims at generating an intracellular environment compatible with biochemical and cell biological processes. Our work provides direct evidence for this importance and points to molecular crowding as a critical parameter to be controlled by cells.

It had been known for some time that Hog1 signaling and nuclear accumulation are delayed at progressively higher stress levels [27,34,42]. We and Miermont et al. [44], which appeared while this work was in preparation, now show that this phenomenon is not restricted to Hog1 signaling but appears to be a more general phenomenon. We observed here, however, that nuclear accumulation of Hog1 appears to be even further delayed compared to Hog1 phosphorylation, i.e. that the otherwise good correlation between Hog1 phosphorylation and Hog1 nuclear appearance does not seem to hold at very high stress levels. Hog1 nuclear accumulation under strong osmostress is also slower than that of Msn2. Hence, it is possible that under high stress levels cytosolic targets are prioritized and Hog1 be temporarily anchored or otherwise engaged in the cytosol. It has been demonstrated that yeast cells can adapt to at least mild osmotic stress without Hog1 nuclear accumulation (but not without Hog1) [37]. This means that cytosolic targets, such as those in cell cycle control [58], protein biosynthesis [59], glycerol/glycolytic metabolism [51,60,61] and glycerol transport [24,25,53], may be prioritized in the early stages of adaptation. Also transport of ions and water may be controlled by Hog1 and hence may play important roles during early adaptation [62,63]. For instance, Hog1-dependent activation of the cytoplasmic Na^+^/H^+^ antiporter Nha1 has been reported to be critical for the re-association of DNA-binding proteins following their initial osmostress-induced dissociation. Probably, higher intracellular ion concentration following osmostress causes dissociation of proteins from chromatin, which is then corrected by the Na^+^/H^+^ antiporter Nha1 and the potassium transporter Tok3, which is also phosphorylated by Hog1[62]. 

It still remains a mystery why cells that have an impaired adaptive system due to reduced or abolished ability to accumulate glycerol, such as *gpd1∆ gpd2∆* cells, also show even more delayed or absent initial responses. This was observed here and previously for Hog1 nuclear accumulation [51]. At the same time, cells “pre-adapted” by a higher basal Hog1 activity (*ptp2∆* mutants) or a higher initial intracellular glycerol level (*fps1∆* mutants) respond faster [27,55,64]. It is not immediately apparent why a cell would display a more sluggish stress response when it has an impaired ability to adapt in the long run; intuitively, the opposite may be expected. The results reported here may provide some explanation for this phenomenon. Cells with a defective osmoregulatory system may from the beginning have a lower free water concentration and hence may display a higher degree of molecular crowding at similar osmostress agent concentrations. The opposite may be true for genetically pre-adapted cells. Volume measurements, however, do not really seem to support this idea but those are a rather crude measure for the actual state of the cytosol because one usually measures cell surface, including the cell wall, and from those data extrapolates to cell volume.

It is not understood how cells sense osmotic changes, i.e. which physical stimulus osmosensor perceive. The S. *cerevisiae* HOG pathway osmosensors Sln1, Msb2 and Hkr1 are located in the plasma membrane and they might somehow sense changes in the spacing or interaction between the plasma membrane and the cell and/or stretching (lipid-lipid or lipid-protein interaction) inside the membrane. The observation that cell volume loss significantly affects protein movements inside the cytoplasm, as reported here and previously [44,65,66] provides scope for other mechanisms of osmosensing. For example, the cellular potassium concentration has been demonstrated as factor stimulating the transport activity of a bacterial osmosensitive solute transporter [67,68]. The ability of proteins to interact with each other is affected both by increased concentrations and slower protein dynamics, due to e.g. molecular crowding, and could potentially serve as a stimulus for an osmosensing mechanism. Since the concentration of molecules inside the cell appears to be a highly important parameter for the functions of the cell such a mechanism would intuitively make sense.

## Supporting Information

Figure S1 **Nuclear accumulation of Hog1 is delayed under severe hyperosmotic stress.** Confocal time lapse images of nuclear localization of Hog1 in wild type cells expressing Hog1-GFP and Nrd1-mCherry following treatment with 400mM and 800mM NaCl to illustrate the delay of Hog1 nuclear localization under severe stress condition. Same data as in Figure 1C but here also including the nuclear marker Nrd1-mCherry.
(TIF)

Figure S2 **Hog1 nuclear accumulation in mutants unable to produce glycerol.** Ratio of Hog1-GFP between nucleus and cytosol as a function of time for different stress levels in the *gpd1∆* *gpd2∆* mutant, which cannot produce/accumulate glycerol. Colors indicate different salt concentrations and symbol sizes represent the standard deviation for each time point. Ca. 60 cells were monitored.
(TIF)

Figure S3 **Expression of Msn2/4-dependent genes under severe osmostress.** Expression levels of the Msn/2Msn4-dependent *ALD2* gene as determined by qPCR. Data represent the *ALD2* expression levels relative to those of the constitutive *ACT1* gene in cells exposed to 400mM and 1,000mM NaCl in wild type, *msn2∆*, and *msn2∆* *msn4∆* mutant cells.
(TIF)
