# Supplementary figures and images for: Osmostress-Induced Cell Volume Loss Delays Yeast Hog1 Signaling by Limiting Diffusion Processes and by Hog1-Specific Effects
Source: PLoS One. 2013 Nov 20;8(11):e80901. doi: 10.1371/journal.pone.0080901 (PMC3835318; doi:10.1371/journal.pone.0080901)

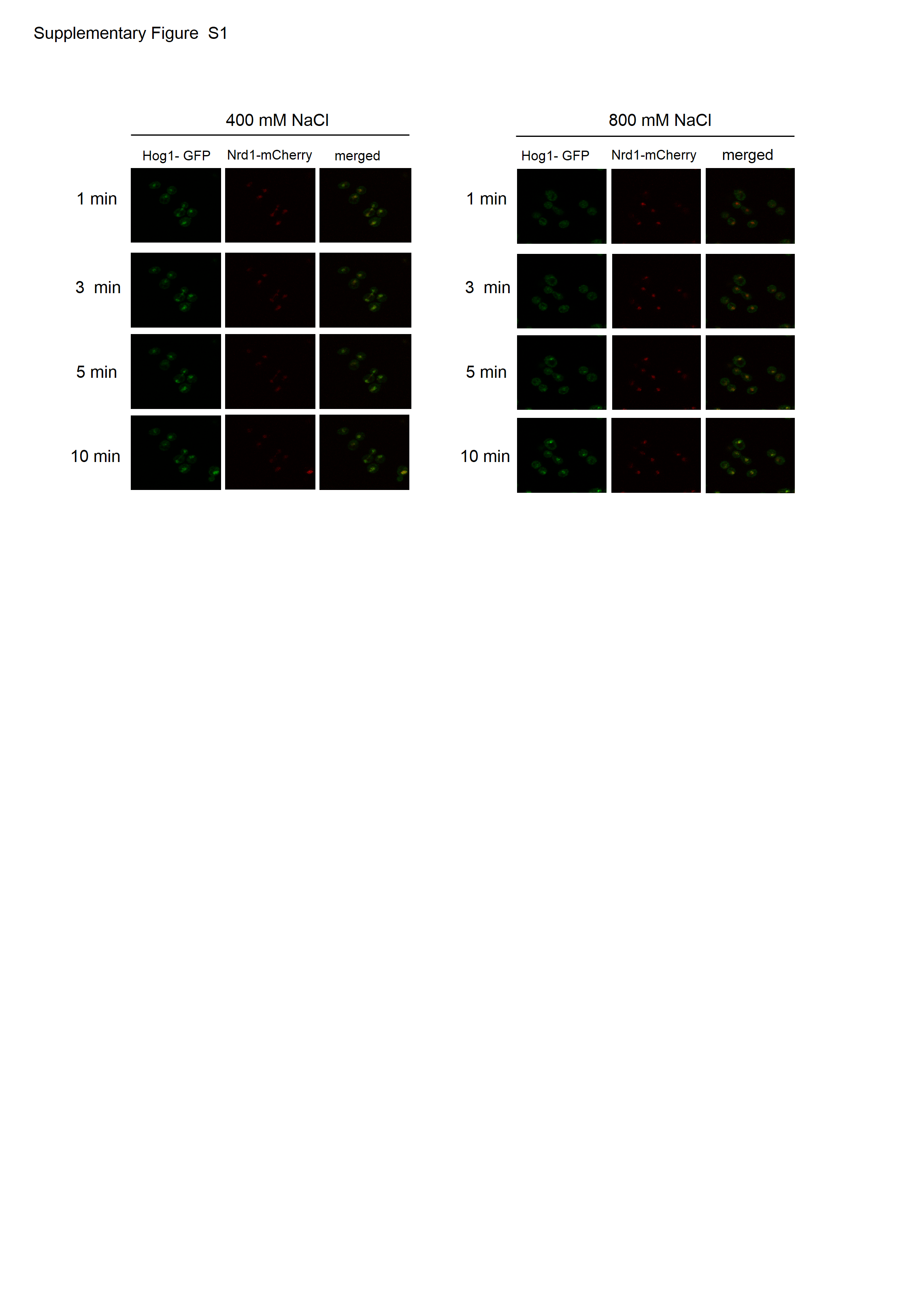

Supplement: Figure S1 — Nuclear accumulation of Hog1 is delayed under severe hyperosmotic stress. Confocal time lapse images of nuclear localization of Hog1 in wild type cells expressing Hog1-GFP and Nrd1-mCherry following treatment with 400mM and 800mM NaCl to illustrate the delay of Hog1 nuclear localization under severe stress condition. Same data as in Figure 1C but here also including the nuclear marker Nrd1-mCherry. (TIF) [file pone.0080901.s001.tif]

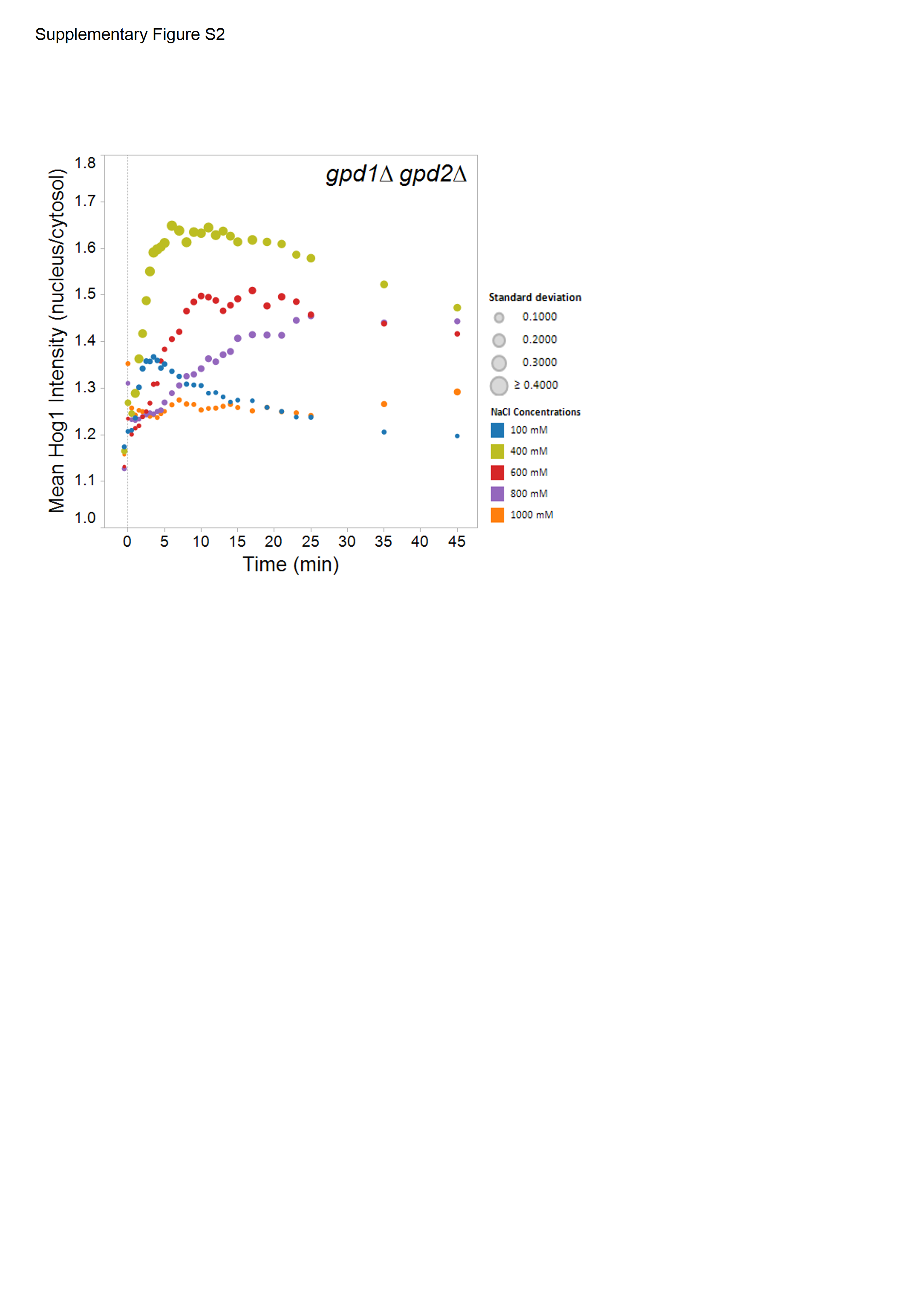

Supplement: Figure S2 — Hog1 nuclear accumulation in mutants unable to produce glycerol. Ratio of Hog1-GFP between nucleus and cytosol as a function of time for different stress levels in the gpd1∆ gpd2∆ mutant, which cannot produce/accumulate glycerol. Colors indicate different salt concentrations and symbol sizes represent the standard deviation for each time point. Ca. 60 cells were monitored. (TIF) [file pone.0080901.s002.tif]

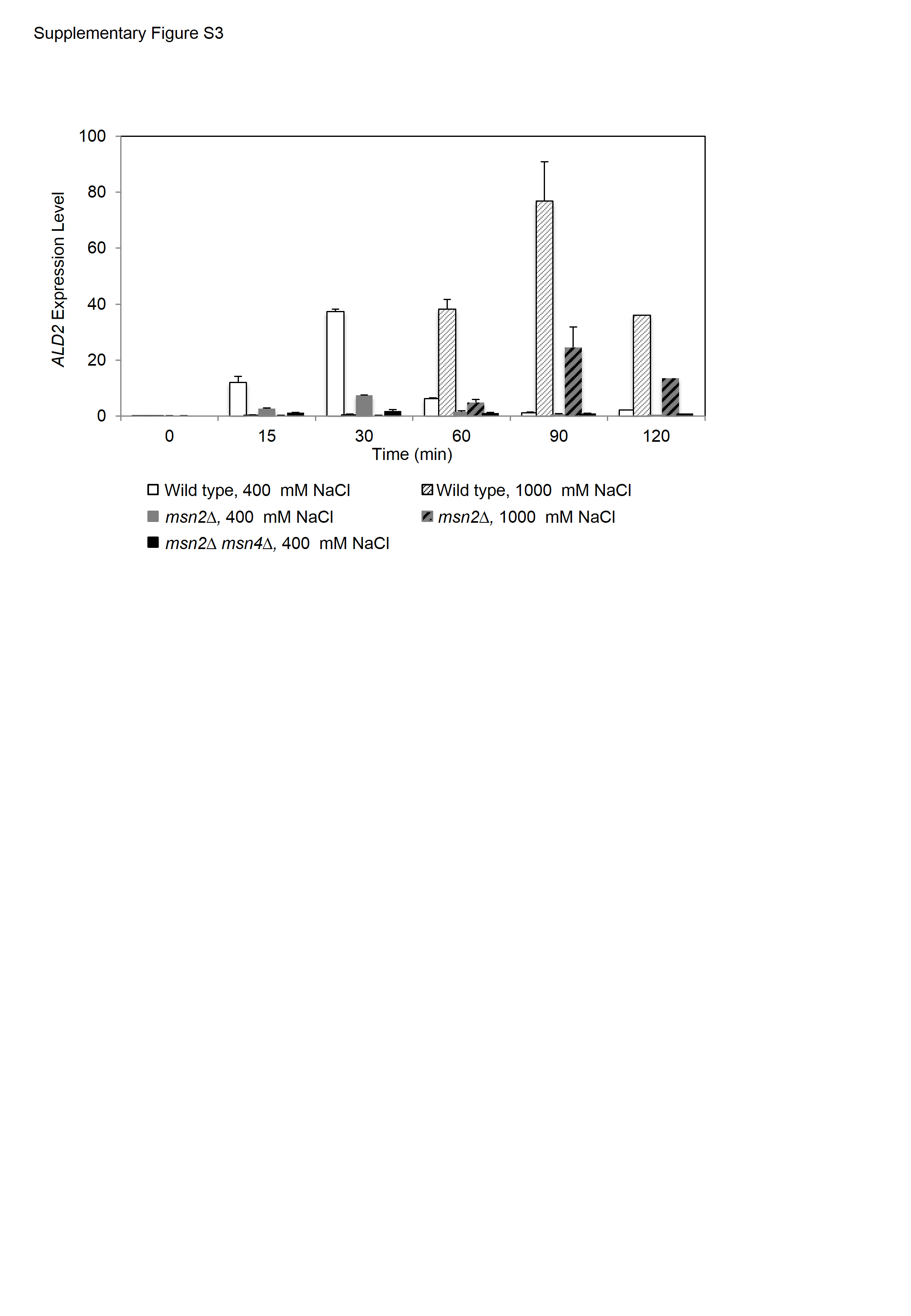

Supplement: Figure S3 — Expression of Msn2/4-dependent genes under severe osmostress. Expression levels of the Msn/2Msn4-dependent ALD2 gene as determined by qPCR. Data represent the ALD2 expression levels relative to those of the constitutive ACT1 gene in cells exposed to 400mM and 1,000mM NaCl in wild type, msn2∆, and msn2∆ msn4∆ mutant cells. (TIF) [file pone.0080901.s003.tif]
